# A Multi-Cancer Mesenchymal Transition Gene Expression Signature Is Associated with Prolonged Time to Recurrence in Glioblastoma

**DOI:** 10.1371/journal.pone.0034705

**Published:** 2012-04-06

**Authors:** Wei-Yi Cheng, Jessica J. Kandel, Darrell J. Yamashiro, Peter Canoll, Dimitris Anastassiou

**Affiliations:** 1 Center for Computational Biology and Bioinformatics, Columbia University, New York, New York, United States of America; 2 Institute for Cancer Genetics, Columbia University, New York, New York, United States of America; 3 Department of Surgery, Columbia University, New York, New York, United States of America; 4 Department of Pediatrics, Columbia University, New York, New York, United States of America; 5 Department of Pathology and Cell Biology, Columbia University, New York, New York, United States of America; 6 Columbia Stem Cell Initiative, Columbia University, New York, New York, United States of America; 7 Department of Electrical Engineering, Columbia University, New York, New York, United States of America; University of Florida, United States of America

## Abstract

A stage-associated gene expression signature of coordinately expressed genes, including the transcription factor Slug (SNAI2) and other epithelial-mesenchymal transition (EMT) markers has been found present in samples from publicly available gene expression datasets in multiple cancer types, including nonepithelial cancers. The expression levels of the co-expressed genes vary in a continuous and coordinate manner across the samples, ranging from absence of expression to strong co-expression of all genes. These data suggest that tumor cells may pass through an EMT-like process of mesenchymal transition to varying degrees. Here we show that, in glioblastoma multiforme (GBM), this signature is associated with time to recurrence following initial treatment. By analyzing data from The Cancer Genome Atlas (TCGA), we found that GBM patients who responded to therapy and had long time to recurrence had low levels of the signature in their tumor samples (*P* = 3×10^−7^). We also found that the signature is strongly correlated in gliomas with the putative stem cell marker CD44, and is highly enriched among the differentially expressed genes in glioblastomas vs. lower grade gliomas. Our results suggest that long delay before tumor recurrence is associated with absence of the mesenchymal transition signature, raising the possibility that inhibiting this transition might improve the durability of therapy in glioma patients.

## Introduction

A multi-cancer stage-associated gene expression signature has recently been identified [1], consisting of a set of genes that are coordinately overexpressed only in samples of cancer that have exceeded a particular stage specific to each cancer type. Table 1 contains a list of the 64 genes corresponding to the top 100 probe sets (as presented in [1]) of the signature. The signature contains numerous epithelial-mesenchymal transition (EMT) markers [2], [3], [4], such as the EMT-inducing transcription factor Slug (SNAI2), as well as COL5A2, FAP, POSTN, COL1A2, COL3A1, FBN1, TNFAIP6, MMP2, GREM1, BGN, CDH11, SPOCK1, DCN, COPZ2, THY1, PCOLCE, PRRX1, PDGFRB, SPARC, INHBA, COL6A2, FN1, ACTA2. However, the signature is also present even in some nonepithelial cancers, such as neuroblastoma and Ewing's sarcoma. In each dataset, the expression level of the co-expressed genes varies in a continuous manner across the samples. In a recent experiment we also confirmed that most of the genes of the signature, including α-SMA, are expressed in some xenografted human cancer cells themselves *in vivo*, but not in the host mouse cells [5]. These results indicate that cancer cells can pass through a mesenchymal transition process to varying degrees ranging from total lack of expression to strong co-expression of the genes of the signature, and therefore the corresponding underlying pathways are activated within the cancer cells, in conjunction with other pathways in the tumor microenvironment providing contextual interactions.

**Table 1:** Genes comprising the Slug-based EMT signature.

Rank	Gene	Rank	Gene
1	COL11A1	33	LOXL2
2	THBS2	34	COL6A3
3	COL10A1	35	MXRA5
4	COL5A2	36	MFAP5
5	INHBA	37	NUAK1
6	LRRC15	38	RAB31
7	COL5A1	39	TIMP3
8	VCAN	40	CRISPLD2
9	FAP	41	ITGBL1
10	COL1A1	42	CDH11
11	MMP11	43	TMEM158
12	POSTN	44	SPOCK1
13	COL1A2	45	SFRP4
14	ADAM12	46	SERPINF1
15	COL3A1	47	DCN
16	LOX	48	C7orf10
17	FN1	49	COPZ2
18	AEBP1	50	NOX4
19	SULF1	51	EDNRA
20	FBN1	52	ACTA2
21	ASPN	53	PDGFRB
22	SPARC	54	RCN3
23	CTSK	55	SNAI2
24	TNFAIP6	56	C1QTNF3
25	HNT	57	COMP
26	EPYC	58	LGALS1
27	MMP2	59	THY1
28	PLAU	60	PCOLCE
29	GREM1	61	COL6A2
30	BGN	62	GLT8D2
31	OLFML2B	63	NID2
32	LUM	64	PRRX1

The average expression level of these 64 genes can be thought of as the expression level of a metagene representing the signature, to which we refer as the “mesenchymal transition metagene.” We hypothesized that this value is associated with clinical data in glioblastoma multiforme (GBM) for which there is rich such data available at The Cancer Genome Atlas (TCGA). We found that there was indeed strong association of the metagene with the phenotype “Days to Tumor Recurrence,” defined as the time period from initial treatment until the date of the diagnosis or recognition of the presence and nature of the return of signs and symptoms of cancer following a period of improvement. Patients who did not experience improvement after therapy have a “null” entry in the corresponding field.

## Methods

For statistical analysis we used the rank sum of the patients with long time to recurrence after ranking the patients in terms of the mesenchymal transition metagene. To evaluate the statistical significance, we calculated the *P* value from its definition using empirical distribution function. In addition, we performed Cox regression between days to tumor recurrence and the expression level of the signature.

We also performed multivariate Cox regression on days to tumor recurrence, using both the expression values of the mesenchymal transition metagene and the four glioblastoma subtypes as covariates.

## Results

**Figure 1:**
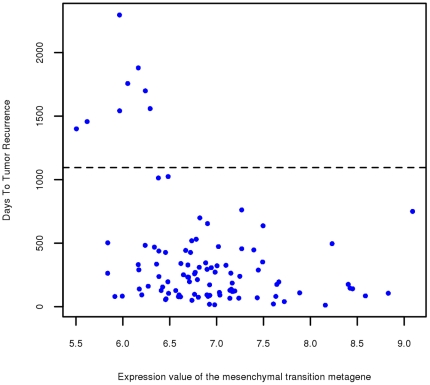
Scatter plot for Days to Tumor Recurrence vs. expression of the mesenchymal transition metagene. Each dot in the scatter plot represents one of the 99 patients for which the “Days to Tumor Recurrence” phenotype has a non-null entry. The horizontal axis measures the average of the RMA-normalized expression levels of the 64 genes shown in Table 1. The vertical axis measures the days to tumor recurrence and the horizontal dotted line is drawn at the 3 year cutoff point.

**Figure 2:**
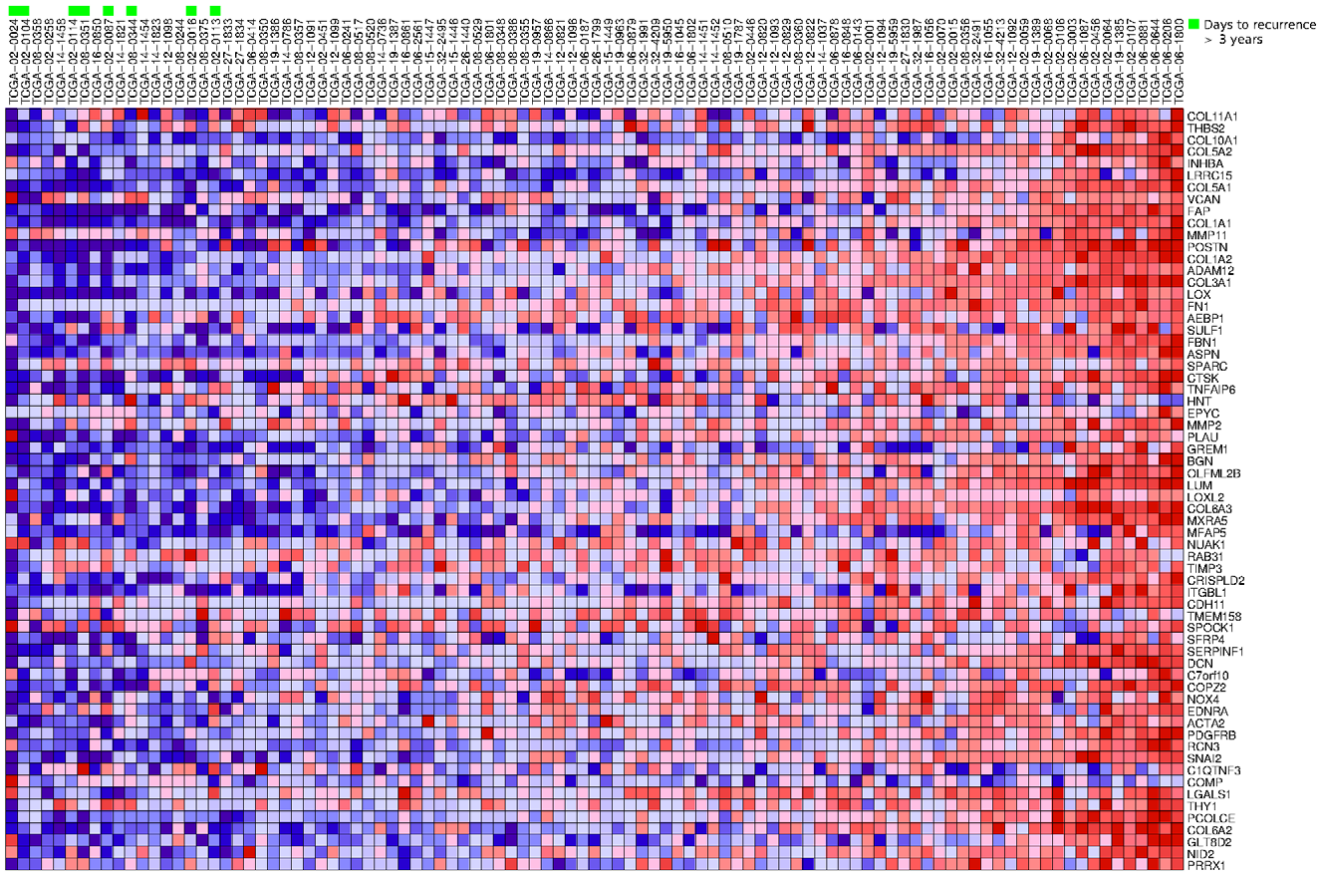
Heat map of the components of the mesenchymal transition metagene in glioblastoma. The 99 samples are ranked in terms of the average expression level of the genes shown in Table 1. The eight patients for which time to recurrence was more than three years are highlighted in green at the 1^st^, 2^nd^, 6^th^, 7^th^, 9^th^, 11^th^, 16^th^, and 18^th^ position, resulting in the rank sum of 70.

Figure 1 shows a scatter plot in which each of the 99 samples for which the “Days to Tumor Recurrence” phenotype has a non-null entry is represented by a dot indicating the expression level of the mesenchymal transition metagene and the number of days to tumor recurrence. The figure reveals that, within the group of patients who experienced improvement after therapy, the eight patients whose tumors recurred more than three years following therapy have very low values of the expression of the metagene. Figure 2 shows a heat map of the 64 genes, where the samples are ranked in terms of the expression of the metagene and the eight patients for which time to recurrence was more than three years are highlighted in green. The rank sum for these eight patients is 1+2+6+7+9+11+16+18 = 70. The rank sum is particularly well suited as a measure of this particular observed aspect of the association of the “Days to Tumor Recurrence” phenotype with the expression of a gene, in which absence of gene expression is required for exceptionally long time to recurrence. The probability of the rank sum being ≤70 due to pure chance is estimated as the relative frequency of such occurrences after randomly permuting the phenotypes ten million times and recalculating the rank sum, concluding that *P* = 3×10^−7^, which is also the probability of finding that the sum of eight randomly picked distinct numbers between 1 and 99 is less than or equal to 70.

**Figure 3:**
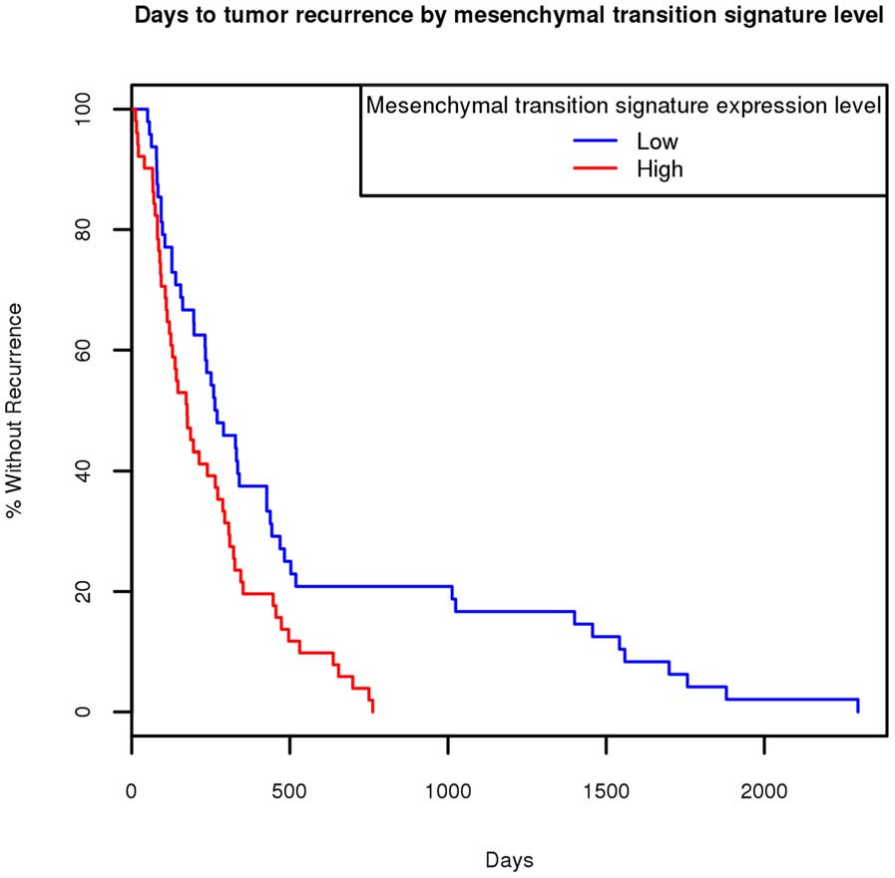
Kaplan-Meier curves comparing samples with high vs. low levels of the mesenchymal transition metagene. The 545 tumor samples were partitioned into two groups of equal size depending on their levels of the mesenchymal transition metagene. Shown are the Kaplan-Meier curves for the corresponding samples with entries in the “Days to Tumor Recurrence” field.

We also separated the entire set of 545 tumor samples into two groups of equal size, containing high vs. low levels of the mesenchymal transition metagene. Within the 99 samples containing a “Days to Tumor Recurrence” field, there were 48 “low level” and 51 “high level” samples. We performed Cox regression between days to tumor recurrence and the expression level of the signature. Figure 3 contains the corresponding Kaplan-Meier survival curves resulting in a clearly seen association with statistical significance of P = 0.0054 using a chi-squared test.

We then used the rank sum metric to identify which, among the individual 64 genes of Table 1 defining the metagene have the best score, expecting that some of them would have rank sum lower than 70. Remarkably, the best scoring gene was COL5A1 with rank sum equal to 78 followed by COL6A2 with rank sum equal to 82. In other words, the score of the metagene is significantly better than that of any of its individual component genes. Even more strikingly, after doing exhaustive search among all 12,042 genes, the top ranked gene (EFEMP2) had rank sum equal to 75, still worse than that (70) of the metagene. These results suggest that the signature identified in [1] comprises a synergistic collection of genes corresponding to a biological mechanism of mesenchymal transition, which, when absent, is associated with increased time period to tumor recurrence in GBM.

Table 2 shows a listing of the top 30 individual genes in terms of their rank sum for the “Days to Tumor Recurrence” phenotype. Nine out of these 30 genes, highlighted in Table 2, are among the 64 genes of Table 1, demonstrating the strong enrichment (*P* = 3×10^−14^) of EMT markers in this unbiased collection of genes associated with the phenotype.

**Table 2:** Top genes in terms of the rank sum for the “Days to Tumor Recurrence” phenotype.

Genes	Rank sum
EFEMP2	75
CD248	78
**COL5A1**	78
IL7R	78
MYH9	81
**COL6A2**	82
FLNC	82
AKAP12	87
TREM1	87
**COL1A2**	88
PSCDBP	88
S100A8	91
CLEC2B	92
GLIPR1	98
**COL6A3**	99
THBD	99
CALD1	102
CD163	102
EFEMP1	102
ENPEP	103
**PCOLCE**	103
TMEM5	103
SDCBP	104
**COL1A1**	105
**LUM**	105
TNC	106
VNN1	106
CARS	107
**FN1**	107
**COL3A1**	108

**Figure 4:**
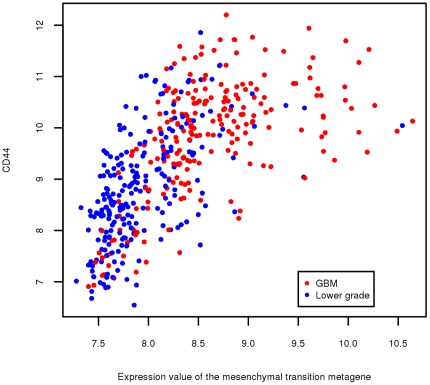
Scatter plot for the expression levels of CD44 vs. the mesenchymal transition metagene. Each dot in the scatter plot represents a glioma sample from the NCI Repository for Molecular Brain Neoplasia Data (Rembrandt) dataset. Dots are color coded red for glioblastomas and blue for lower grade gliomas. Expression levels are RNA normalized.

While all cases in the TCGA dataset have been diagnosed as glioblastoma, the delayed recurrence in these eight cases is more a characteristic of lower grade gliomas. Therefore, we investigated whether lower grade gliomas are also characterized by lower levels of the signature by analyzing the NCI Repository for Molecular Brain Neoplasia Data (Rembrandt) dataset, which included gene expression from both glioblastoma as well as various types of lower grade gliomas. Table 3 demonstrates that, indeed, there is strong enrichment (seven of the 64 genes in Table 1 are among the top-ranked 30 differentially expressed genes, *P* = 10^−13^). Furthermore, we found strong correlation between the expression levels of the metagene and the cancer stem cell marker CD44 (*P* = 5×10^−56^ based on fitting Pearson correlation to t-distribution). Figure 4 shows the corresponding scatter plot. Recent studies have shown that high levels of CD44 are expressed in cancer stem cells isolated from several different types of tumors [6], although this concept is still in evolution, and CD44 is also expressed in a variety of other cell types. CD44 has been found in a cell population enriched for glioma stem cells [7]. It is also widely expressed in glioblastoma, and increased levels are associated with glioma progression and resistance to therapy [8].

**Table 3:** Top differentially expressed gene in glioblastomas vs. lower grade gliomas.

Probe Set ID	Gene Symbol	Fold Change
209395_at	CHI3L1	10.36
202718_at	IGFBP2	6.07
210809_s_at	**POSTN**	5.77
201666_at	TIMP1	5.70
1556499_s_at	**COL1A1**	5.69
215076_s_at	**COL3A1**	5.59
202404_s_at	**COL1A2**	5.17
206157_at	PTX3	4.90
201012_at	ANXA1	4.87
202237_at	NNMT	4.82
211527_x_at	VEGFA	4.73
221898_at	PDPN	4.65
202912_at	ADM	4.65
215446_s_at	**LOX**	4.43
202345_s_at	FABP5	4.41
226517_at	BCAT1	4.30
203729_at	EMP3	4.14
202018_s_at	LTF	4.05
227697_at	SOCS3	3.96
211981_at	COL4A1	3.64
209156_s_at	**COL6A2**	3.62
201505_at	LAMB1	3.59
226237_at	226237_at	3.59
236028_at	IBSP	3.57
201744_s_at	**LUM**	3.53
225681_at	CTHRC1	3.52
203645_s_at	CD163	3.51
211964_at	COL4A2	3.49
201110_s_at	THBS1	3.44
208949_s_at	LGALS3	3.42

Analysis of gene expression data has resulted in classification into various subtypes of glioblastomas [9], [10], also present in lower grade gliomas [11], with distinct features, each of which is characterized by the presence of particular genes. Interestingly, CD44 was found enriched in the mesenchymal subtypes in all these cases. The feature of our current results, however, is that the mesenchymal transition signature used in this paper reflects a biological process applicable to multiple cancer types, as it was derived by analyzing its presence in many different cancers [1], as opposed to using classification methods on glioma samples alone to identify subtypes. Furthermore, the association with the phenotype is found in the absence, rather than the presence, of the signature.

To confirm that the observed association with the “Days to Tumor Recurrence” phenotype is more related to the presence of the mesenchymal transition signature, rather than to the classification into a mesenchymal subtype, we performed multivariate Cox regression on days to tumor recurrence, using both the expression values of the mesenchymal transition metagene and the four subtypes [9] as covariates. The subtype variable is a categorical variable with four types (Mesenchymal, Classical, Neural and Proneural). To infer the samples whose subtypes were not given in the original paper, we performed a ten-nearest neighbor imputation based on the signature genes of the four subtypes as given in [9]. The result shows that the mesenchymal transition metagene expression variable is the only significant covariate (with *P* = 0.049), while the rest of the categorical variables did not pass the significance level of 0.05 (the minimum was 0.160 for the Mesenchymal subtype), demonstrating that the “Days to Tumor Recurrence” phenotype is most significantly associated with the mesenchymal transition signature. The results of Cox regression are shown in Table 4.

**Table 4:** Multivariate Cox regression using GBM subtypes as covariates.

	Coefficient	SE(Coef)	Hazard ratio (95% CI)	*P* value
**Mesenchymal transition metagene**	0.307	0.156	1.359 (1.001–1.844)	0.049
**Subtype** *				
Overall			1.000	
Mesenchymal	0.284	0.203	1.328 (0.892–1.977)	0.160
Classical	−0.141	0.175	0.868 (0.617–1.223)	0.419
Neural	0.080	0.255	1.083 (0.657–1.785)	0.750
Proneural	−0.222	0.203	0.801 (0.537–1.193)	0.270

Likelihood ratio test = 12.3, degrees of freedom = 4, *P* value = 0.0153.

* The table shows all four linear contrasts between individual subtype mean log-hazard ratios and overall mean log-hazard ratio. In practice there are three contrasts in each Cox regression regardless of which contrast is chosen as the noncoding group. We used two separate Cox regressions to produce the results in the table.

To further compare directly the mesenchymal transition signature with that of the Mesenchymal subtype of [9], we created a metagene for the latter so that we can evaluate its association with the “Days to Tumor Recurrence” phenotype as measured by the rank sum. This was created using the gene list as described in the supplementary information of the paper, available at http://tcga-data.nci.nih.gov/docs/publications/gbm_exp. Specifically, in the associated data file containing the expression values and subtype calls for the Core TCGA samples using the unified scaled data, there are 216 genes labeled as mesenchymal. These genes were ranked in terms of their power to represent the mesenchymal phenotype, as determined by the differences between each gene's mesenchymal centroid component and the centroid component of the remaining subtypes, which can also be regarded as the log-fold change between the gene's mean value in the mesenchymal subtype and the gene's overall mean [12] (as quoted in the data file containing the ClaNC840 gene list and centroids). Based on that ranking, we selected the top 64, so that the sizes of the two metagenes to be compared are identical. The value of the rank sum was 142 (it would have been 151 if using all 216 genes). This should be compared with the corresponding value of 70 of the mesenchymal transition metagene and with the other entries of individual genes in Table 2. These results further confirm that the observed association with days to tumor recurrence is due to the multi-cancer mesenchymal transition signature, which has the remarkable property that the corresponding metagene has lower rank sum than any individual gene.

## Discussion

Because gliomas are not epithelial cancers, and the signature has also been found in other nonepithelial cancers, such as neuroblastoma and Ewing's sarcoma, the signature represents a more general biological process of mesenchymal transition, applicable to all solid cancers that we tried. Indeed, when the set of genes of Table 1 are the input for Gene Set Enrichment Analysis (GSEA) [13] against the Molecular Signatures Database (MSigDB), there are many results with *P* value exactly equal to “zero,” corresponding to genes expressed in higher-stage samples from many cancer types, such as nasopharyngeal, head and neck, urothelial, lymphomas, etc. Such cancer types had not participated in any way whatsoever in the derivation of the signature. This remarkable validation of the signature by pointing to all kinds of cancer types in MSigDB suggests that the signature may reflect a universal biological mechanism of mesenchymal transition present in the invasive stage of all solid cancers including glioblastoma. Analysis of related datasets suggests that there are multiple affected pathways comprising a particularly complex biological mechanism that appears to reactivate embryonic developmental programs. Indeed, when analyzing the 64-gene signature against MSigDB Gene Ontology biological process datasets, the top five results were all related to development (skeletal, organ, multicellular organismal, system, anatomical structure). The prominent GO cellular component was extracellular matrix, and the prominent GO molecular function was collagen binding.

It has recently been suggested that “stemness” in tumor cells (characterized by the ability to both self-renew as well as generate differentiated descendants) may be intimately interconnected with passing through an EMT. For example, EMT in some models was found to generate cells with properties of stem cells [14], [15], [16], [17], [18]. Notably, it has been shown that stem-like cells isolated from human breast cancer co-express high levels of CD44 and high levels of mesenchymal markers, including Slug [14]. Furthermore, inducing EMT in immortalized human mammary epithelial cells leads to high levels of CD44 expression in the mesenchymal-like cells [14]. Drug resistance has also been linked to the presence of cancer stem cells [16], [18], [19], [20], supporting the notion that cancer stem cells may be responsible for recurrence after therapeutic intervention. Therefore, and given the strong correlation of the mesenchymal transition signature with CD44, one possible explanation for the absence of the mesenchymal transition signature in patients with exceptionally long time to recurrence may be due to a corresponding lack of stemness in the cancer cells of these patients making it more unlikely for the cancer to recur following treatment. An alternative explanation for the observed association may be provided by the transformation towards a more mesenchymal phenotype [21].

Although there are several EMT-inducing transcription factors [22], some of which are also found occasionally upregulated in the mesenchymal transition signature characterized by the genes in Table 1, Slug is the only one found consistently upregulated. It was also the only such transcription factor upregulated in our experimental xenografts [5]. Slug has also recently been found to be associated with invasiveness in glioma [23], consistent with the results presented here. Furthermore, when we ranked all genes in terms of their correlation (using the measure of mutual information [24]) of their expression with that of Slug in the 99 samples that we analyzed here, we found that, remarkably, the top eight entries (COL6A3, COL3A1, LUM, COL5A1, COL1A2, COL6A2, COL1A1, PCOLCE) were all genes included in both Tables 1 as well as Table 2, further supporting the hypothesis that Slug might be a master regulator of the biological mechanism responsible for the signature. It was recently found [25], however, that induced overexpression of the transcription factor Twist in glioblastoma leads to increased invasiveness and expression of several of the genes in Table 1, including Slug, suggesting that Twist may play a causative role for the mesenchymal transition in glioblastoma.

The same signature was also found to be predictive of neoadjuvant therapy in breast cancer - see, e.g. additional file 6 of [1], in which 7 of 8 samples in the cluster on the left side of the heat map (with low levels of the signature) had good response to therapy, while 12 out of 14 samples in the second cluster (with high levels of the signature) were resistant.

The observations that (a) all GBM patients with exceptionally long time to recurrence had extremely low levels of the mesenchymal transition gene signature, and (b) the mesenchymal transition signature is strongly enriched among the genes underexpressed in lower grade gliomas as compared to glioblastomas, suggest that targeting the underlying biological mechanism might supply a novel approach for adjuvant treatment of gliomas. Further, the ability to precisely identify components of the gene signature provides unique opportunities for identifying potential targets for such treatment.
